# Vitamin D Supplementation Does Not Enhance Gains in Muscle Strength and Lean Body Mass or Influence Cardiorespiratory Fitness in Vitamin D-Insufficient Middle-Aged Men Engaged in Resistance Training

**DOI:** 10.3390/nu16193356

**Published:** 2024-10-02

**Authors:** Lauri Savolainen, Saima Timpmann, Martin Mooses, Evelin Mäestu, Luule Medijainen, Märt Lellsaar, Kristi Tiimann, Anneli Piir, Mihkel Zilmer, Eve Unt, Vahur Ööpik

**Affiliations:** 1 Institute of Sport Sciences and Physiotherapy, University of Tartu, 18 Ülikooli St., 50090 Tartu, Estonia; laurisavolainen@outlook.com (L.S.); saima.timpmann@ut.ee (S.T.); martin.mooses@ut.ee (M.M.); evelin.maestu@ut.ee (E.M.); luule.medijainen@ut.ee (L.M.); mart.lellsaar@gmail.com (M.L.); 2 Dermatology Clinic, Tartu University Hospital, 31 Raja St., 50417 Tartu, Estonia; kristi.tiimann@gmail.com; 3 Department of Biochemistry, Institute of Biomedicine and Translational Medicine, University of Tartu, 50090 Tartu, Estonia; anneli.piir@ut.ee (A.P.); mihkel.zilmer@ut.ee (M.Z.); 4 Department of Cardiology, Institute of Clinical Medicine, University of Tartu, 50090 Tartu, Estonia; eve.unt@kliinikum.ee; 5 Department of Sport Medicine and Rehabilitation, Institute of Clinical Medicine, University of Tartu, 50090 Tartu, Estonia; 6 Sport Medicine and Rehabilitation Clinic, Tartu University Hospital, 1a Puusepa St., 50406 Tartu, Estonia

**Keywords:** testosterone, cortisol, growth hormone, parathormone, insulin, HOMA-IR, inflammatory status

## Abstract

Background: This study checked whether vitamin D (Vit-D) supplementation improves the efficacy of resistance training (RT) in terms of increasing muscle strength and lean body mass (LBM), and influencing cardiorespiratory fitness (VO_2_max) in Vit-D-deficient middle-aged healthy men. Methods: Participants (*n* = 28) were quasi-randomly assigned to one of two groups, which, in a double-blind manner, supplemented their diet daily with either Vit-D (8000 IU; VD) or placebo (PLC) during participation in a 12-week supervised RT program. Results: During the intervention, serum Vit-D concentrations increased 2.6-fold (*p* < 0.001) in the VD group, while no changes occurred in the PLC group. Muscle strength gains (*p* < 0.001) as measured in seven exercises performed on RT equipment and increases (*p* < 0.001) in LBM were similar in the two groups. Total fat mass, percent total fat, and percent android fat decreased (*p* < 0.05) to a similar extent in both groups, but there was no change in VO_2_max in either group. Conclusions: In conclusion, in healthy Vit-D-insufficient middle-aged men engaged in resistance training, Vit-D supplementation increases serum 25(OH)D levels but does not enhance gains in muscle strength and LBM, or decreases in fat mass and fat percentage, and does not affect cardiorespiratory fitness.

## 1. Introduction

Vitamin D (Vit-D) is considered a critical and important micronutrient for overall health [1,2], with much attention given to its importance in bone and skeletal muscle health [3,4]. Vit-D can also affect the function of the lungs, heart, and blood vessels [5,6,7], the production of pro-inflammatory and anti-inflammatory cytokines [8,9,10], and participate in the regulation of insulin sensitivity [11].

The bioactive form of Vit-D acts through specific nuclear receptors (VDR) [8,12,13], which have been identified in most tissues of the human body [14,15,16]. In skeletal muscle, Vit-D exerts both genomic and non-genomic effects [17,18]. In human myoblasts, Vit-D has been shown to stimulate protein synthesis [19]. In older women, Vit-D supplementation increased intramyonuclear VDR concentration and muscle fiber size [20].

However, recent literature reviews on the effects of Vit-D supplementation on muscle strength, an important indicator of muscle health [21], have led to conflicting conclusions. Meta-analyses have shown that Vit-D supplementation has no effect on muscle strength [22], can increase it [23], can only increase muscle strength in the lower limbs [24,25], or can increase handgrip strength depending on the daily dose of Vit-D, duration of treatment, and baseline serum 25(OH)D level [26]. A systematic review [27] concluded that supplementation with Vit-D3 increases muscle strength, but supplementation with Vit-D2 is ineffective. Of note, the majority of these papers [22,23,24,27] included studies of relatively young athletic populations. Only Abshirini et al. [26] and Muir and Montero-Odasso [25] focused on middle-aged and older subjects.

Undoubtedly, the most effective strategy for increasing muscle strength and mass is resistance training (RT) [28,29,30], but it is less clear whether combining Vit-D supplementation with RT improves its outcomes. Recent meta-analysis revealed that Vit-D supplementation had an additive effect to RT in increasing muscle strength of the lower limbs in older adults [31], but this conclusion was based on the results of only three studies. A more recent study [32] in elderly persons with or without chronic obstructive pulmonary disease showed the effectiveness of a 13-week RT program in increasing muscle mass and strength but found no additional effect of concomitant vitamin D supplementation on RT-associated changes in muscle mass or function.

Maximal oxygen uptake (VO_2_max) is often referred to as cardiorespiratory fitness and is considered an objective measure of health [33,34]. The functional state of the lungs, heart, blood vessels and skeletal muscles, together with the oxygen transport capacity of the blood, are the major determinants of an individual’s VO_2_max [35]. VDR is expressed in all these organs, i.e., lungs, heart, blood vessels, and skeletal muscle [5,6,7,17]. Vit-D can promote erythropoiesis and hemoglobin synthesis [36], and affect the binding affinity of oxygen to hemoglobin [37]. Furthermore, Vit-D may be crucial for mitochondrial oxidative phosphorylation capacity [38]. Therefore, it is not surprising that an independent robust association has been observed between serum Vit-D levels and VO_2_max in adults over an age range of 20–73 years [39,40].

However, it is not clear whether Vit-D supplementation is effective at increasing VO_2_max. A recent literature review [41] concluded that Vit-D supplementation can significantly improve VO_2_max in elite athletes, but this claim was based on the results of only two studies. Research in athletes of varying levels and in recreationally active youth has yielded inconclusive results, showing no effect [42,43] or a marginal positive influence [44] of Vit-D supplementation on VO_2_max. In some of the studies in young men [45,46] that reported a positive effect of Vit-D supplementation on cardiorespiratory fitness, VO_2_max was not actually measured but indirectly calculated. In elderly patients with chronic obstructive pulmonary disease participating in a rehabilitation program, Hornikx et al. [47] observed significantly greater improvements in VO_2_max and inspiratory muscle strength as a result of Vit-D supplementation compared to placebo.

Muscle mass and strength [48], as well as VO_2_max [49], declined with increased age and may also lead to decreased quality of life, loss of independence, and disability in the elderly. Thus, measures that can forestall declines in muscle strength and VO_2_max may be important considerations for middle-aged adults that may help maintain better health status and reduce the risk of substantial decline in quality of life in older age. The literature cited above suggests that RT, combined with Vit-D supplementation, may be an effective intervention in this context, but the available data are limited and inconclusive, particularly for the middle-aged population. Therefore, the main objective of this study was to assess whether systematic RT, combined with Vit-D supplementation, is more effective in terms of increasing muscle strength and lean body mass (LBM) than the same RT program without Vit-D supplementation in Vit-D-insufficient middle-aged healthy men. The secondary objective was to elucidate whether Vit-D supplementation during participation in an RT program affects cardiorespiratory fitness in the same men.

## 2. Materials and Methods

### 2.1. Participants

**Figure 1:**
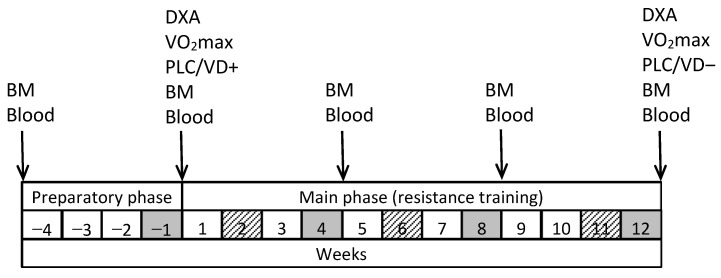
Study design. Arrows indicate time points for measuring body mass (BM), body composition (DXA), maximal oxygen uptake (VO_2_max), venous blood sampling (Blood), and beginning (PLC/VD+) and end (PLC/VD−) of placebo or vitamin D supplementation. Grey and striped cells indicate the weeks during which muscle strength was assessed and 4-day food diaries were completed, respectively.

Middle-aged and older men (aged > 50 years) were sent invitations to participate in the study via emails and phone calls that were obtained through personal contacts and various organizations’ mailing lists. The initial inclusion criterion was the absence of a chronic disease that would preclude participation in systematic physical training. Additional criteria were non-participation in competitive sports or resistance training programs, and non-use of Vit-D supplements in the past year. The total number of men who expressed willingness to participate in the study and met these inclusion criteria was 31. At the first meeting with the research team, each potential participant received detailed information about the purpose of the study, the procedures related to participation in it, and the time required for this. The men who chose to participate in the study then provided relevant verbal and written informed consent and donated the baseline venous blood sample. During the subsequent 4-week preparatory phase (Figure 1), one man dropped out of the study due to injury and two withdrew for personal reasons. All the remaining 28 men went through the 12-week main phase of the study, participating in at least 80% of the planned resistance training sessions, performing all the planned physical tests, and donating all the necessary blood samples. The age, height, body mass, and body mass index (mean ± SD) of these 28 men at the beginning of the preparatory phase of the study were 58.8 ± 4.4 years, 182.9 ± 6.3 cm, 90.03 ± 15.26 kg, and 26.9 ± 4.0 kg/m^2^, respectively.

### 2.2. Design and General Organization of the Study

The study was carried out according to a double-blind, placebo controlled, quasi-randomized protocol, and it was divided into a 4-week preparatory and a 12-week main phase (Figure 1). The entire 16-week study took place from January to April, i.e., during the winter–spring period of the year, when the prevalence of Vit-D insufficiency and deficiency is high in Estonia [50,51].

In the preparatory phase, participants were familiarized with RT equipment, taught to perform strength exercises technically correctly, and instructed on documenting their regular diet and loading the information into a web-based platform, Nutridata. This period also aimed to adapt participants to a consistent training schedule, equalize the training levels of the participants, and to address the neural and learning adjustments that occur in the first weeks of starting systematic RT [52]. At the end of the preparatory phase, the participants were divided into two groups: one receiving a placebo (PLC) and the other Vit-D supplements (VD). This was done by arranging participants in ascending order of body mass and then assigning them to alternate groups. No significant differences in age, height, body mass, body mass index, or Vit-D status were found between the groups at the beginning of the study (Table 1). According to the definitions proposed by Pludowski et al. [53], the Vit-D status of the participants at this stage of the study was classified as deficient (9 men with serum 25(OH)D concentrations <50 nmol/L), insufficient (16 men with serum 25(OH)D concentrations ≥50 and <75 nmol/L), or sufficient (3 men with serum 25(OH)D concentrations 75–125 nmol/L). Since men with Vit-D deficiency and insufficiency accounted for 32% and 57%, respectively, and given that according to Heaney [54] the distinction between Vit-D deficiency and insufficiency may not be crucial, we describe the participants in this study generally as “Vit-D-insufficient men”.

In the main phase of the study, participants attended supervised RT sessions three times a week and consumed either Vit-D supplements or placebo daily. At the conclusion of each 4-week training cycle, muscle strength assessments were conducted. Participants were instructed to log their dietary intake for four consecutive days on three separate occasions using the Nutridata web-based platform in the main phase (Figure 1).

The participants underwent a maximal oxygen uptake (VO_2_max) test to measure their aerobic capacity and body composition assessment, both before and after the main phase of the study (Figure 1). Body mass was measured, and blood samples were taken five times, starting before the beginning of the preparatory phase, and continuing with 4-week intervals until the conclusion of the main phase (Figure 1).

### 2.3. Supervised Resistance Training and Assessment of Muscle Strength

The RT program included 7 exercises [55] performed on special gym equipment: seated row, knee extension, biceps curl, triceps push-down, lateral pull-down (Precor, Woodinville, WA, USA), leg press (David, Helsinki, Finland), and chest press (Technogym, Cesena, Italy). All training sessions in both the preparatory and main phases were supervised by a member of the research team and took place at the University of Tartu Sports Club. Training frequency was two sessions per week for the first two weeks of the preparatory phase, increasing to three sessions per week from the third week onward until the end of the study. Training sessions were scheduled in the morning or evening to accommodate the individual daily routines of participants. Each session began with a 10–15 min light warm-up performed on a 200-m indoor running track, cycle, or rowing ergometer. The sequence of exercises within a session was flexible, except for the knee extension and leg press, which required an upper body exercise to be performed between them. Each exercise’s concentric and eccentric phases took about 1–2 s. Rest periods between sets ranged from 1 to 2 min, and the interval between different exercises was set at 3 min [56]. Participants were required to complete all sets of one exercise consecutively before moving on to the next exercise. The number of sets and repetitions varied throughout the study (Table 2). In the preparatory phase, initial training weights for every exercise were determined by the supervisor, and in the main phase training weights were adjusted based on a percentage of each individual’s one-repetition maximum (1RM), as outlined in Table 2. This way the weights aligned with each participant’s personal progress. 1RMs were estimated from five-repetition maximums (5RMs), as described by Baechle and Earle [57]. These 5RM tests were conducted at weeks −1, 4, 8, and 12 (Figure 1), and were used to set individual training weights for every exercise for the next 4-week training cycle. The 5RM testing protocol involved progressively increasing weights to determine the maximum weight lifted five times correctly. A detailed description of the 5RM assessment procedure is provided in our previous publication [58]. The 5RM values were ascertained in order of lateral pull-down, triceps push-down, leg press, and chest press during the first training session of the RM testing week. In the second session, seated row, knee extension, and biceps curl were assessed in that order. In addition to the 5RM tests, participants performed one remaining set of exercises in which no tests were performed in a given testing session. In the third training session of the RM testing week, participants performed one set of each exercise, as shown in Table 2. Regular RT sessions lasted approximately one hour. The first two sessions of 1RM testing weeks lasted approximately 45–55 min (testing plus a reduced number of training exercises).

### 2.4. Dietary Supplementation

Throughout the main phase of the study, participants were instructed to administer gelatine capsules containing either vitamin D_3_ or placebo. The Vit-D and placebo capsules were identical, they could not be distinguished by appearance, and participants were provided with them in a double-blind manner. The product codes for Vit-D and placebo capsules were ST45851 and ST47202, respectively (Diafarm A/S, Vejle, Denmark). The daily dose of supplemental Vit-D was 8000 IU. In the main phase, following each training session, all participants ingested 50 g of a whey-based dietary supplement (Whey 80, Elite Fitness OY, Helsinki, Finland). Each 50 g serving of this supplement provided 40 g of pure whey protein, 2.5 g of carbohydrates, 3.5 g of fats, and 200 kcal of energy. At the end of each workout, the training supervisor personally handed out the supplement, instructing participants to dissolve the whey powder in about 0.5 L of water and consume it within 30 min. Standardization of protein intake during early post-workout recovery was deemed important because consuming protein in this period influences muscle protein synthesis [59,60], and post-workout changes in muscle protein synthesis are linked to the degree of muscle hypertrophy that manifests as a result of several weeks RT [61].

### 2.5. Monitoring of Dietary Intake

At weeks 2, 6, and 11 of the main phase (Figure 1), participants recorded their food intake over four consecutive days on the Nutridata platform. Each recording period included one non-training day, typically during the weekend, when participants were not engaged in their regular work. Participants granted a member of the research team access to their Nutridata entries, allowing this individual to conduct detailed dietary analyses on both an individual and group basis. Throughout the study, participants were instructed to maintain their usual dietary patterns and to avoid consuming any supplements except those provided by the research team member, i.e., Vit-D or placebo and whey protein.

### 2.6. Assessment of Body Size and Composition

The height of the subjects was determined to an accuracy of 5 mm using a stadiometer (Seca bodymeter 206, Seca GmbH, Hamburg, Germany). Body mass (BM) without clothing was measured following an overnight fast on five occasions throughout the study (Figure 1) to the precision of 1 g using an electronic scale (CH3G-150I Combics, Sartorius AG, Goettingen, Germany). Before and at the end of the main phase (Figure 1) participants underwent dual-energy X-ray absorptiometry (DXA) body composition analysis (Hologic Discovery W model; Hologic, Inc., Marlborough, MA, USA). Measurements included the lean mass of the torso, arms, and legs, alongside the mass and percentage of android fat, and the total fat mass and percentage.

### 2.7. Assessment of Maximal Oxygen Uptake

Participants took a graded exercise test on a motorized treadmill both before and after the main phase of the study (Figure 1) to assess their maximum oxygen uptake and related parameters. The equipment used included a Viasys/Jaeger LE300 C motorized treadmill (Viasys Healthcare GmbH, Hoechberg, Germany), a MasterScreen CPX breath-by-breath metabolic system (Viasys Healthcare GmbH, Hoechberg, Germany), and a heart rate monitor (Polar Electro Oy, Kempele, Finland). An incremental walking test began with a 1.5% incline and a constant speed of 6.0 km/h. The treadmill’s incline was increased by 3.5% every three minutes while maintaining the speed, until the participant could no longer continue. The highest average oxygen uptake over a 30-s period towards the end of the test was recorded to determine the maximal oxygen uptake (VO_2_max). Achievement of VO_2_max was confirmed if the participant had reached a respiratory exchange ratio (RER) greater than 1.00 and a heart rate exceeding 90% of their predicted maximum based on age, per the criteria set by Davis et al. [62].

### 2.8. Blood Sampling and Analyses

During the study, participants had five venous blood samples drawn at specified times: at the beginning of the preparatory phase, after the preparatory phase concluded, and after the 4th, 8th, and 12th week of the main phase (Figure 1). All the samples were collected after two days of rest, following a roughly 12-h overnight fast, either on a Monday or Tuesday morning. The blood was drawn into 5-mL Vacutainer serum tubes, which were left to clot for 10 min at room temperature. The samples were then centrifuged for another 10 min at 3000 rpm (2000× *g*) at a temperature of 4 °C using an Eppendorf 5804R centrifuge (Eppendorf AG in Hamburg, Germany). Post-centrifugation, the samples were kept at 4 °C until analysis, and the serum specimens for the measurement of cytokine levels were maintained at −25 °C.

All five blood samples were analyzed for serum 25(OH)D. Concentrations of various biomarkers—testosterone, growth hormone (GH), insulin-like growth factor-1 (IGF-1), cortisol, parathormone (PTH), calcium (Ca), ionized Ca, urea, creatine kinase (CK), glucose, ferritin, and insulin—were measured in the samples taken just before the main phase and right after the 8th and 12th week (Figure 1). These analyses were conducted at the United Laboratories of Tartu University Hospital. In the blood sample taken before and after the main phase (Figure 1), serum cytokine levels for interleukin 1α (IL-1α), interleukin 1β (IL-1β), interleukin 4 (IL-4), interleukin 6 (IL-6), interleukin 8 (IL-8), interleukin 10 (IL-10), tumor necrosis factor alpha (TNF-α), and monocyte chemoattractant protein 1 (MCP-1) were measured. The cytokine analysis was performed at the Institute of Biomedicine and Translational Medicine of the University of Tartu.

An IDS-iSYS Multi-Discipline Automated Analyzer (Immunodiagnostic Systems Limited, Copenhagen, Denmark) was used to measure serum concentrations of 25(OH)D, IGF-1, and GH using the chemiluminescence immunoassay method. The electrochemiluminescence immunoassay (ECLIA) on the Cobas 6000 analyzer (Roche Diagnostics GmbH, Tokyo, Japan) was used to determine levels of testosterone, cortisol, PTH, CK, and insulin. For serum Ca and urea concentration measurements, the same Cobas 6000 analyzer was employed, using the photometrical NM-BAPTA method and a kinetic test with urease and glutamate dehydrogenase, respectively. Ionized Ca was measured with an ion-selective electrode method on a Prime ES analyzer (Roche, Mannheim, Germany). Serum glucose levels were quantified using the hexokinase enzymatic method on a Roche/Hitachi Cobas 6000 c501 analyzer (Roche Diagnostics GmbH, Mannheim, Germany). The homeostasis model assessment of insulin resistance (HOMA-IR) index was calculated using the formula: HOMA-IR = (serum glucose (mmol/L) × serum insulin (μU/mL))/22.5 [63].

The Evidence Investigator Cytokine and Growth Factors High-Sensitivity Array based on the sandwich chemiluminescent immunoassay, version V1.4.1 (RANDOX Laboratories Ltd., Crumlin, UK), was used for the simultaneous quantitative detection of cytokines.

### 2.9. Statistical Analysis

Data were analyzed using Statistica software (Version 13.5.0.17; TIBCO Software Inc., Palo Alto, CA, USA). All data were checked for normal distribution using the Kolmogorov–Smirnov test, which revealed that IL-1α and IL-1β were not normally distributed and were therefore log-transformed. A two-way repeated analysis of variance ANOVA with a between-factor of group (VD vs. PLC) and within-factor of time was used to evaluate the differences within and between the groups. If a significant main effect or interaction occurred, Tukey’s honestly significant difference post hoc analysis was used to locate differences between the means. Partial η-squared (η_p_^2^) is reported as a measure of effect size. A small effect was reported for η_p_^2^ > 0.01, a medium effect for η_p_^2^ > 0.06, and a large effect for η_p_^2^ ≥ 0.14. The mean values of different parameters registered at a single time point were compared using the Student’s t test for independent variables. Significance was set at *p* < 0.05 level. Data are presented as means ± SD. We are not aware of previous studies in which individuals similar to our participants (age, sex, and Vit-D status), and the potential combined effects of daily high-dose (8000 IU) Vit-D supplementation and three-month RT on muscle strength, body composition, and VO_2_max (primary outcomes), have been assessed. Therefore, the software G*Power^®^, version 3.1.9.2, was used to calculate the ANOVA a priori test for repeated measures with two-group and time interactions (*p* < 0.05; power of 80%), resulting in a minimum total sample of 24 participants.

## 3. Results

The energy and nutrient intake of the participants is shown in Table 3. As the compliance of many participants with the requirement to report daily energy and nutrients intake was low, only reliable data that were limited to six men from both groups for weeks 2 and 6 are presented. No main effects of group or time, or group-by-time interaction (in all cases *p* > 0.05), occurred for any of the dietary parameters measured.

**Figure 2:**
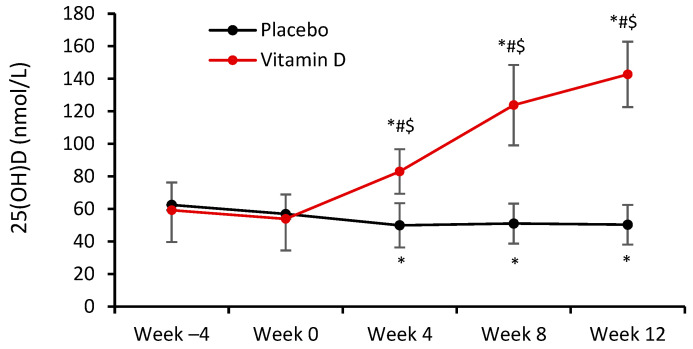
Serum 25(OH)D concentrations. Data are presented as mean ± SD, *n* = 14 in placebo, and *n* = 14 in vitamin D group. Significantly different (*p* < 0.05): * from Week −4; ^#^ from previous week; ^$^ from placebo group.

For serum 25(OH)D levels, significant main effects of group (F = 47.91; η_p_^2^ = 0.648) and time (F = 113.29; η_p_^2^ = 0.813), and a significant group-by-time interaction (F = 170.25; η_p_^2^ = 0.867), were observed (*p* < 0.0001 in all cases). At weeks −4 and 0, serum 25(OH)D concentrations were similar in the two groups (Figure 2). At week 4, i.e., four weeks after starting Vit-D and placebo administration, serum 25(OH)D levels were significantly higher in the VD group than in the PLC group (83.0 ± 13.7 nmol/L vs. 49.9 ± 13.6 nmol/L, respectively; *p* = 0.0002). In the VD group, serum 25(OH)D continued to rise throughout the rest of the main phase of the study, remaining consistently significantly higher than in the PLC group, reaching a level of 142.7 ± 20.1 nmol/L at week 12. In the PLC group, there was a significant (*p* = 0.010) decrease in serum 25(OH)D from week −4 to week 4, after which 25(OH)D remained consistently low from 51.0 ± 12.3 to 50.3 ± 12.2 nmol/L until week 12.

**Figure 3:**
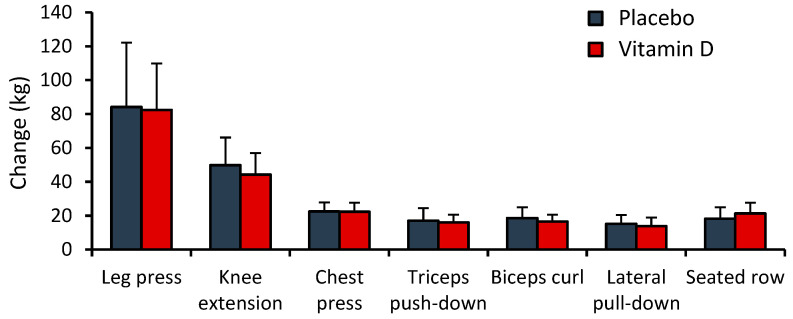
Changes in 1-repetition maximum during 12-week supplementation and resistance training period. Data are presented as mean ± SD, *n* = 14 in placebo, and *n* = 14 in vitamin D group.

Compliance with the RT protocol was high (91.5% on average) and similar in the two groups (91% in PLC and 92% in VD). A significant main effect of time was observed for 1RM for all seven exercises: leg press (F = 133.0; η_p_^2^ = 0.8423), knee extension (F = 158.90; η_p_^2^ = 0.864), chest press (F = 351.12; η_p_^2^ = 0.931), triceps push-down (F = 132.29; η_p_^2^ = 0.836), biceps curl (F = 209.50; η_p_^2^ = 0.890), lateral pull-down (F = 143.11; η_p_^2^ = 0.846), and seated row (F = 176.44; η_p_^2^ = 0.872) (*p* < 0.0001 in all cases) (Table 4). However, no significant main effect of group or group-by-time interaction was observed for 1RM for any of the exercises (*p* > 0.05 in all cases). In both groups, the greatest increase in 1RM was seen in knee extension (69.3% and 83.6% in VD and PLC, respectively), and the smallest change in triceps push-down (23.3% and 26.4% in VD and PLC, respectively) (Figure 3).

Significant main effects of time were found for body mass (F = 4.68; *p* = 0.005; η_p_^2^ = 0.153), total fat mass (F = 18.05; η_p_^2^ = 0.410), total fat percentage (F = 48.88; η_p_^2^ = 0.653), android fat percentage (F = 29.62; η_p_^2^ = 0.532), total lean mass (F = 78.34; η_p_^2^ = 0.751), trunk lean mass (F = 29.19; η_p_^2^ = 0.529), arms lean mass (F = 33.86; η_p_^2^ = 0.566), and legs lean mass (F = 28.20; η_p_^2^ = 0.520) (*p* < 0.001 in all cases) (Table 5). No significant main effect of time was observed for android fat mass (F = 3.059; *p* = 0.092; η_p_^2^ = 0.105). There were no main effects of group or group-by-time interactions for any of the body composition parameters (*p* > 0.05 in all cases). Over the 12-week RT program, both the PLC and VD groups exhibited significant increases in total lean mass, trunk lean mass, arms lean mass, and legs lean mass, as well as decreases in total fat mass, fat percentage, and android fat percentage (*p* < 0.05 in all cases). However, there were no significant between-group differences in the extent of changes in any body composition parameter (*p* > 0.05 in all cases).

A significant main effect of time was found for relative VO_2_max (mL/min/kg) levels (F = 5.158; *p* = 0.032; η_p_^2^ = 0.171), with no significant main effect of group (*p* = 0.473) or group-by-time interaction (*p* = 0.839) (Table 6). A small (2.3%) overall decrease in relative VO_2_max occurred between weeks 0 and 12 (*p* = 0.031). For absolute VO_2_max (L/min), there was no significant main effect of group (*p* = 0.533) or time (*p* = 0.146), nor a significant group-by-time interaction (*p* = 0.551). No significant main effect of group (*p* = 0.841) or time (*p* = 0.062), nor group-by-time interaction (*p* = 0.445), were observed for the RER. A significant group-by-time interaction occurred for peak HR (F = 4.979; *p* = 0.035; η_p_^2^ = 0.166), with no significant main effects of time (*p* = 0.406) or group (*p* = 0.301). No significant main effects of group or time, or group-by-time interactions, were observed for ventilation (VE) and breathing frequency (BF) (*p* > 0.05 in all cases) (Table 6).

A significant group-by-time interaction occurred for parathormone levels (F = 8.768; *p* = 0.0005; η_p_^2^ = 0.252), with no main effect of time or group (*p* > 0.05 in both cases) (Table 7). In the PLC group, parathormone levels increased by 22.8% from week 0 to week 12 (*p* = 0.008). A significant main effect of time (F = 8.357; *p* = 0.0007; η_p_^2^ = 0.243), but not that of group (*p* = 0.916), occurred for cortisol. An overall decrease of 16.1% (*p* = 0.002) in cortisol levels was evident from week 0 to week 12, but there was no significant group-by-time interaction for cortisol (*p* = 0.787). No main effects of group or time, nor group-by-time interactions, were observed for IGF-1, testosterone, growth hormone, and insulin levels (*p* > 0.05 in all cases) (Table 7).

No significant main effects of group and time, or group-by-time interactions, were observed for serum levels of IL-1α, IL-1β, IL-4, IL-6, IL-8, IL-10, TNF-α, and MCP-1, or for the IL-10/TNF-α ratio (*p* > 0.05 in all cases; Table 8).

There were no significant main effects of group or time, and no group-by-time interactions, for hemoglobin and glucose concentrations (in all cases *p* > 0.05; Table 9). A significant main effect of time (F = 7.595; *p* = 0.0013; η_p_^2^ = 0.226), but not that of group (*p* = 0.837) or group-by-time interaction (*p* = 0.984), occurred for serum ferritin levels (Table 9). Overall serum ferritin levels decreased from 194.4 ± 116.3 μg/L at week 0 to 167.6 ± 105.2 μg/L at week 12, i.e., by an average of 13.8% (*p* = 0.011). There was a significant group-by-time interaction (F = 3.532; *p* = 0.036; η_p_^2^ = 0.120), but no significant main effects of group or time (in both cases *p* > 0.05) for HOMA-IR (Table 9).

No main effects of group or time, or group-by-time interactions, were observed for serum-ionized calcium and calcium concentrations, and creatine kinase activity (in all cases *p* > 0.05; Table 10). A significant main effect of time (F = 3.824; *p* = 0.028; η_p_^2^ = 0.128) and group-by-time interaction (F = 4.704; *p* = 0.013; η_p_^2^ = 0.153) with no significant main effect of group (*p* = 0.555) occurred for serum urea levels. In the VD group only, a significant increase in serum urea levels was observed in week 8 (*p* = 0.005) and week 12 (*p* = 0.036), compared to week 0 (Table 10).

## 4. Discussion

The primary objective of this study was to assess whether systematic RT combined with Vit-D supplementation is more effective in terms of increasing muscle strength and LBM than the same RT program without Vit-D supplementation in Vit-D-insufficient healthy middle-aged men. The results show that over the course of 12 weeks of RT, 1RM increased significantly and to a similar extent in all seven exercises in both the VD and PLC groups. During the same period, serum 25(OH)D increased approximately 2.6-fold in the VD group, but remained stably low in the PLC group. These data are consistent with previous findings of a positive impact of RT on muscle strength in middle-aged men [64,65,66], but indicate that Vit-D supplementation does not potentiate the effects of RT in these age and sex groups.

Four previous studies [32,56,67,68] have also shown that Vit-D supplementation has no additional effect on RT in increasing muscle strength in middle-aged and elderly individuals. However, in two studies the participants were exclusively [68] or mostly (90%) [67] elderly women. It should also be noted that these two studies only measured maximal isometric quadricep strength [68], or quadricep and handgrip strength [67]. Given that testosterone can influence the RT-induced gains in muscle strength [69,70], and that Vit-D supplementation can increase serum testosterone levels [71,72], sex differences in the effect of Vit-D supplementation on the efficacy of RT cannot be excluded a priori. It is also relevant to evaluate the potential effect of combined RT and Vit-D supplementation on strength gains in different muscle groups, as they may respond differently to Vit-D supplementation [24]. Nevertheless, our data presented here on middle-aged men agree with those obtained in studies of older women [67,68], and show that RT improves muscle strength, but Vit-D supplementation does not increase the efficacy of RT. In addition, our muscle strength data from seven different exercises show no differences between muscle groups in terms of response to the Vit-D supplementation in middle-aged men.

Agergaard et al. [56] investigated the possible effects of Vit-D supplementation during 12 weeks of systematic RT on the quadricep muscle strength gain in somewhat older healthy men (60–75 years) than our participants (52–66 years) and found no additive effect of Vit-D on either muscle hypertrophy or muscle strength. However, they observed improved muscle quality (increased muscle strength/cross sectional area ratio) in the Vit-D-supplemented group compared to the placebo group. In healthy subjects and patients with chronic obstructive pulmonary disease (age 56–77 years; 46% men), Vit-D supplementation over 13 weeks of RT did not affect maximal muscle strength, assessed by unilateral knee extension and leg press, or bilateral chest press [32]. Our data showing the absence of an additive effect of Vit-D supplementation on muscle strength gains during RT are generally consistent with those of these researchers [32,56], but extend to much more muscle groups in a slightly younger age group of men.

In our participants, 12 weeks of systematic RT had no effect on body weight but induced significant positive health-related changes in body composition, consisting of significant decreases in total fat mass, total fat percentage, and android fat percentage. At the same time, significant increases occurred in total lean mass, trunk lean mass, and appendicular lean mass.

However, all changes in body composition were of a similar extent in the VD and PLC groups, indicating that Vit-D supplementation did not increase the efficacy of RT. Mølmen et al. [32] reported increases in total lean mass, total fat mass, and visceral fat mass, which are in good agreement with our findings, showing the efficacy of RT with no additive effect of Vit-D supplementation.

One of our considerations for why Vit-D might improve the efficacy of RT in terms of muscle strength gains in middle-aged men was the vitamin’s potential positive effect on testosterone levels [71,72]. However, in our participants, Vit-D supplementation had no effect on serum testosterone or other hormones (insulin, growth hormone, IGF-1, cortisol) that may affect muscle protein metabolism and muscle function. Similar findings have been reported by other researchers [32,67].

The secondary objective of this study was to elucidate whether systematic RT or Vit-D supplementation during participation in an RT program affects cardiorespiratory fitness in Vit-D-insufficient healthy middle-aged men. Our results show no effect of 12 weeks of RT with or without concomitant Vit-D supplementation on absolute VO_2_max. Overall, a small (2.3%) decrease in relative VO_2_max occurred over 12 weeks of RT.

We considered the assessment of the potential effect of interventions applied in this study on VO_2_max important for several reasons. First, skeletal muscle oxidative capacity that is among the factors determining VO_2_max is dependent on mitochondrial function [73], and systematic RT may lead to positive mitochondrial adaptations [74,75,76]. Second, in middle-aged-to-elderly subjects, systematic RT may produce significant increases in VO_2_max that are as large as in the case of aerobic training [77], or slightly smaller [78,79]. Third, VDR is expressed in all human organs, the function of which may influence VO_2_max [5,6,7,17]. Vit-D can promote erythropoiesis and hemoglobin synthesis [36], and affect the binding affinity of oxygen to hemoglobin [37]. Finally, Vit-D may be crucial for mitochondrial oxidative phosphorylation capacity [38]. Nevertheless, 12 weeks of RT with or without concomitant Vit-D supplementation did not influence blood hemoglobin levels or absolute VO_2_max in our participants. At the same time, there was a small but statistically significant decrease in relative VO_2_max, probably due to a tendency to gain weight.

An overall significant 13.8% decline in serum ferritin levels across the 12-week supplementation and RT period indicates a decrease in the body’s iron stores [80] and is consistent with our previous observation in young Vit-D-deficient men who completed a similar RT program with concomitant Vit-D supplementation [81]. However, serum ferritin levels <35 μg/L, indicating an iron-deficient state [80], occurred only in two participants at week 0, and in one of these two participants also at weeks 8 and 12. Both men belonged to the VD group. Considering that hemoglobin concentrations were constantly >130 g/L in all participants, i.e., there were no manifestations of anemia, and that iron deficiency without anemia does not affect VO_2_max [80], it seems unlikely that low iron status masked the potential effect of Vit-D supplementation on VO_2_max in our participants. Moreover, Shoemaker et al. [82] demonstrated significant training-induced increases in VO_2_max, despite 73% decreases in serum ferritin levels in young men. The reasons why training loads can lead to a decrease in ferritin levels are not entirely clear, and they may differ in different situations. It has been speculated that the improvement of various aspects of performance against the background of a decrease in serum ferritin may reflect the training tolerance of athletes [83] and physiological adaptation reactions, for example, at the level of intensification of the synthesis of iron-containing enzyme proteins [84].

Previous studies on middle-aged adults [75] and elderly men [76] have demonstrated mitochondrial adaptations as a result of 10 and 6 weeks of RT, respectively, but VO_2_max was not measured by these researchers. In studies showing a significant positive effect of RT on VO_2_max [77,78], the duration of systematic RT was 6 months, i.e., twice as long as in our participants. Thus, the RT program in our participants may have been too short-term to induce measurable changes in VO_2_max.

In our participants, there were no between-group differences or changes across time in physiological parameters measured during VO_2_max tests (respiratory exchange ratio, maximal heart rate, maximal breath frequency, maximal pulmonary ventilation). These findings are consistent with the lack of meaningful changes in VO_2_max. Interestingly, Kujach et al. [42] observed increases in maximal breath frequency and maximal lung ventilation due to Vit-D supplementation in college-age males.

The bioactive form of Vit-D is known to inhibit and stimulate the production of pro- and anti-inflammatory cytokines, respectively [8,9,10]. In young healthy Vit-D-deficient men, 12 weeks of RT with concomitant Vit-D supplementation improved the inflammatory status, as reflected by an increase in the serum IL-10/TNF-α ratio [81]. In middle-aged men in the present study, RT and Vit-D supplementation did not exhibit this kind of interaction, and no changes were observed in any of the measured cytokine levels. The reasons for the discrepancy in the results of these two studies remain unclear, but it can be assumed that they may be partly related to the fact that our middle-aged men had higher pre-intervention Vit-D status compared to young participants in the previous [81] study. On the other hand, the recent findings of Silva et al. [85] show that 12 weeks of RT per se, i.e., without Vit-D supplementation, may have an anti-inflammatory effect, including a strong tendency to improve the IL-10/TNF-α ratio, in middle-aged and elderly patients with chronic obstructive pulmonary disease (COPD). The difference between our data and those of Silva et al. [85] regarding the anti-inflammatory effect of RT in middle-aged and elderly people may be at least partially explained by the fact that COPD is an inflammatory disease [86], but our subjects were healthy men.

Chronic Vit-D deficiency is associated with insulin resistance [11]. Six-month Vit-D supplementation in obese Vit-D-insufficient adolescents [87] and 12 weeks of RT in obese middle-aged men [88] improved insulin resistance, which was reflected in a significant decrease in the HOMA-IR index. In our normal weight middle-aged men, 12 weeks of RT with or without Vit-D supplementation did not affect serum glucose and insulin levels, or HOMA-IR. Our results are consistent with those of three previous studies in overweight and obese young adults with varying Vit-D status [89], overweight and obese middle-aged older adults with sufficient Vit-D status and type 2 diabetes [90], and normal weight young Vit-D-deficient men [81] who participated in a similar 12-week RT program with concomitant Vit-D supplementation.

In our VD group participants, the daily dose of supplemental Vit-D was 8000 IU, but the tolerable upper intake level of Vit-D established for adults is 4000 IU per day [91]. On the other hand, a daily dose of 10,000 IU is considered the lowest-observed-adverse-effect-level of Vit-D intake [91], and the position of The Endocrine Society is that a daily intake of this amount may be necessary to treat Vit-D deficiency [92]. Nevertheless, Vit-D oversupply may lead to serious health problems, including the formation of kidney stones, calcification of soft tissues, and vasculature [53,93]. Hypercalcemia is a sensitive marker of the potential harmful effects of Vit-D [92,94,95]. It is widely accepted that hypercalcemia induced by Vit-D intoxication usually only occurs at serum 25(OH)D concentrations above 375 nmol/L, and is very rare [92,95,96]. In our VD group participants, serum calcium levels remained unchanged throughout the study period. The highest 25(OH)D concentrations in their serum occurred at week 12 and were in the range of 120.7–188.1 nmol/L, i.e., well below the critical level.

In our PLC group participants, serum 25(OH)D levels did not increase during the RT and placebo supplementation periods, and were in the range of 23.5–63.1 nmol/L at week 12. Vit-D insufficiency may lead to secondary hyperparathyroidism that has negative implications for cardiovascular and bone health [97,98]. In the PLC group, but not in the VD group, serum parathormone levels increased during the 12 weeks of RT and supplementation, but still remained within the physiologically normal range. Thus, the data discussed in the last two sections show that neither high-dose Vit-D supplementation nor placebo posed a risk to the health of our participants.

Our participants donated blood samples always in the morning after two resting days. Under these conditions, fasting serum urea concentrations below 7.5 mmol/L and moderate levels of creatine kinase activity suggest that our participants tolerated training loads well and started each consecutive training week in a well-recovered state [99].

The main strengths of our study are the supervised RT and dietary supplementation program, and high compliance rate of participants with both requirements. On the other hand, the main limitation of the study was the low compliance of the subjects with the guidelines for reporting daily energy and nutrient intake for three 3-day periods of the 12-week intervention. Therefore, we were only able to analyze reliable dietary data of six participants for weeks 2 and 6, but we abandoned the analysis of the 11-week dataset altogether, due to its low quality. Although all participants verbally confirmed that their usual diet did not change during their participation in the study, and the data of the men who followed the corresponding instructions correctly for at least half the duration of the study confirm that, our data remain incomplete in this regard. Nevertheless, we are not aware of any circumstances that directly or indirectly would indicate that changes in habitual diet could have masked the possible effects of Vit-D supplementation on the outcomes of our study.

## 5. Conclusions

In conclusion, in healthy Vit-D-insufficient middle-aged men engaged in a 12-week RT program, Vit-D supplementation increases serum 25(OH)D levels and avoids increases in serum parathormone concentration, but does not enhance RT-induced gains in muscle strength and LBM, or decreases in fat mass and fat percentage. A 12-week RT with or without a concomitant Vit-D supplementation does not affect cardiorespiratory fitness, serum pro- and anti-inflammatory cytokine levels, or HOMA-IR, an index of insulin resistance.

## Figures and Tables

**Table 1:** Anthropometric characteristics and serum vitamin D concentrations in the two groups of participants at the beginning of the main phase of the study.

Variable	Placebo (*n* = 14)	Vitamin D (*n* = 14)
Age (years)	58.3 ± 4.7	59.2 ± 4.2
Height (cm)	181.9 ± 5.6	183.9 ± 7.0
Body mass (kg)	90.01 ± 15.54	90.05 ± 15.56
BMI (kg/m^2^)	27.1 ± 4.0	26.6 ± 4.0
25(OH)D (nmol/L)	62.5 ± 13.8	59.2 ± 19.5

Data are presented as mean ± SD. BMI, body mass index.

**Table 2:** Distribution of training loads during the preparatory and main phases of the study.

Phase	Week	SPW	Sets	Reps	Training Loads (Weight Used)
Preparatory	−4	2	2	15–20	Chosen by supervisor
	−3				
	−2	3	3	12–15	
	**−1**	**3**	**1**	**12–15**	
Main	1	3	3	12–15	60–75% of the 1RM measured at week −1
	2				
	3				
	**4**	**3**	**1**	**12–15**	
	5	3	3	8–12	60–75% of the 1RM measured at week 4
	6				
	7				75–85% of the 1RM measured at week 4
	**8**	**3**	**1**	**8–12**	
	9	3	3	8–12	75–85% of the 1RM measured at week 8
	10				
	11				
	**12**	**3**	**1**	**8–12**	

1RM test weeks are presented in bold; 1RM, 1-repetition maximum; SPW, number of training sessions per week; Sets, number of sets of each exercise performed during each training session; Reps, number of repetitions per one set of each exercise.

**Table 3:** Daily energy and nutrient intake.

Variable	Group	Week 2	Week 6
Energy intake (kcal)	PLC	2309 ± 259	2241 ± 431
	VD	1953 ± 533	1942 ± 412
Protein (%)	PLC	17.6 ± 2.7	15.9 ± 1.7
	VD	16.9 ± 2.2	16.2 ± 2.5
Protein (g/kg) *	PLC	1.15 ± 0.26	1.03 ± 0.34
	VD	1.05 ± 0.41	0.98 ± 0.31
Fat (%)	PLC	37.6 ± 3.3	39.5 ± 6.7
	VD	38.3 ± 7.0	37.7 ± 4.2
Fat (g/kg)	PLC	1.10 ± 0.21	1.10 ± 0.24
	VD	1.04 ± 0.36	1.06 ± 0.44
Carbohydrates (%)	PLC	41.7 ± 6.5	42.0 ± 6.7
	VD	43.1 ± 8.3	43.9 ± 4.2
Carbohydrates (g/kg)	PLC	2.81 ± 0.91	2.75 ± 0.97
	VD	2.73 ± 1.10	2.70 ± 0.86
Vitamin D (μg) **	PLC	5.45 ± 3.74	4.27 ± 2.15
	VD	9.28 ± 5.65	6.28 ± 3.89
Calcium (mg)	PLC	908 ± 309	933 ± 330
	VD	744 ± 316	643 ± 222

Data are presented as mean ± SD, *n* = 6 in PLC (placebo), and *n* = 6 in VD (vitamin D) group. * The amounts of protein in the table do not include the 40 g of whey which was consumed only on the training days after each workout. ** For both groups, the table only shows the amounts of vitamin D that the subjects consumed with daily food and not in the form of a dietary supplement.

**Table 4:** One-repetition maximum (1RM) strength (kg) during 12-week supplementation and resistance training period.

Variable	Group	Week 0	Week 4	Week 8	Week 12
Leg press	PLC	192.9 ± 33.0	228.5 ± 31.7 *	249.5 ± 35.9 *^#^	277.2 ± 45.6 *^#^
	VD	198.7 ± 45.1	231.6 ± 42.0 *	249.4 ± 40.8 *	281.1 ± 48.5 *^#^
Knee extension	PLC	59.6 ± 20.4	77.7 ± 24.1 *	92.9 ± 25.3 *^#^	109.5 ± 19.2 *^#^
	VD	63.9 ± 23.1	79.9 ± 26.0 *	94.4 ± 24.1 *^#^	108.1 ± 24.6 *^#^
Chest press	PLC	57.8 ± 12.8	66.0 ± 12.7 *	72.3 ± 13.4 *^#^	80.3 ± 13.9 *^#^
	VD	62.3 ± 15.2	69.4 ± 14.9 *	76.6 ± 16.0 *^#^	84.5 ± 17.3 *^#^
Triceps push-down	PLC	64.6 ± 10.0	70.5 ± 10.1 *	75.8 ± 10.4 *^#^	81.7 ± 10.4 *^#^
	VD	69.1 ± 10.8	75.5 ± 10.7 *	80.2 ± 11.3 *^#^	85.1 ± 12.5 *^#^
Biceps curl	PLC	52.3 ± 9.1	60.8 ± 10.4 *	66.0 ± 11.5 *^#^	70.9 ± 12.1 *^#^
	VD	53.7 ± 10.6	59.9 ± 10.6 *	64.9 ± 10.5 *^#^	70.2 ± 10.8 *^#^
Lateral pull-down	PLC	56.6 ± 12.4	60.9 ± 11.6 *	65.0 ± 12.0 *^#^	71.7 ± 11.6 *^#^
	VD	57.8 ± 11.3	61.0 ± 11.0	65.6 ± 10.4 *^#^	71.7 ± 10.5 *^#^
Seated row	PLC	63.7 ± 12.1	70.1 ± 11.7 *	75.8 ± 12.5 *^#^	81.9 ± 13.3 *^#^
	VD	64.6 ± 13.2	72.4 ± 14.0 *	78.8 ± 15.5 *^#^	85.8 ± 16.0 *^#^

Data are presented as means ± SD, *n* = 14 in PLC (placebo), and *n* = 14 in VD (vitamin D) group. Significantly different (*p* < 0.05): * from Week 0; ^#^ from previous week.

**Table 5:** Body mass and body composition during 12-week supplementation and resistance training period.

Variable	Group	Week 0	Week 12	Change
Body mass (kg)	PLC	90.01 ± 15.54	90.47 ± 14.72	0.46 ± 1.87
	VD	90.05 ± 15.56	90.98 ± 15.14	0.93 ± 1.27
Total fat mass (kg)	PLC	23.81 ± 7.66	22.59 ± 6.95 *	–1.23 ± 1.60
	VD	24.42 ± 7.80	23.44 ± 7.90 *	–0.98 ± 1.11
Total fat (%)	PLC	26.5 ± 4.5	25.0 ± 4.4 *	–1.5 ± 1.2
	VD	27.2 ± 4.9	25.7 ± 4.8 *	–1.5 ± 1.1
Android fat mass (kg)	PLC	2.18 ± 0.82	2.12 ± 0.75	–0.06 ± 0.25
	VD	2.34 ± 0.99	2.25 ± 0.97	–0.09 ± 0.21
Android fat (%)	PLC	32.0 ± 6.0	30.0 ± 6.5 *	–2.0 ± 1.9
	VD	33.1 ± 7.2	31.3 ± 6.9 *	–1.7 ± 1.7
Total lean mass (kg)	PLC	61.41 ± 8.45	63.24 ± 8.34 *	1.82 ± 1.07
	VD	60.76 ± 8.38	62.80 ± 8.15 *	2.04 ± 1.24
Trunk lean mass (kg)	PLC	30.85 ± 4.39	31.70 ± 4.30 *	0.86 ± 0.75
	VD	30.96 ± 4.37	31.80 ± 3.96 *	0.85 ± 0.91
Arms lean mass (kg)	PLC	7.12 ± 1.26	7.45 ± 1.23 *	0.33 ± 0.24
	VD	7.01 ± 1.13	7.50 ± 1.10 *	0.49 ± 0.47
Legs lean mass (kg)	PLC	20.10 ± 3.02	20.69 ± 3.02 *	0.59 ± 0.44
	VD	19.40 ± 2.93	19.98 ± 2.99 *	0.59 ± 0.71

Data are presented as means ± SD, *n* = 14 in PLC (placebo), and *n* = 14 in VD (vitamin D) group. * Significantly different from Week 0 (*p* < 0.05).

**Table 6:** VO_2_max, respiratory exchange ratio, peak heart rate, ventilation, and breath frequency during VO_2_max test.

Variables	Placebo (*n* = 14)		Vitamin D (*n* = 14)	
	Week 0	Week 12	Week 0	Week 12
VO_2_max (mL/min/kg)	40.23 ± 6.10	39.26 ± 6.10	38.48 ± 6.15	37.67 ± 5.81
VO_2_max (L/min)	3.60 ± 0.55	3.52 ± 0.57	3.45 ± 0.52	3.42 ± 0.51
RER	1.11 ± 0.05	1.13 ± 0.05	1.12 ± 0.05	1.13 ± 0.05
HR (beats/min)	162.2 ± 13.1	165.5 ± 13.9	169.6 ± 12.9	168.1 ± 10.7
VE (L/min)	125.7 ± 23.0	128.9 ± 21.2	118.2 ± 21.6	121.7 ± 28.0
BF (times/min)	39.9 ± 9.3	40.1 ± 6.5	36.3 ± 5.4	38.1 ± 6.9

Data are presented as mean ± SD. RER, respiratory exchange ratio; HR, heart rate; VE, ventilation; BF, breath frequency.

**Table 7:** Serum hormone concentrations during 12-week supplementation and resistance training period.

Variable	Group	Week 0	Week 8	Week 12
Parathormone (pmol/L)	PLC	4.29 ± 1.29	4.88 ± 1.70	5.27 ± 1.80 *
	VD	4.90 ± 1.17	4.38 ± 0.97	4.33 ± 1.05
Testosterone (nmol/L)	PLC	18.6 ± 5.6	18.6 ± 6.1	17.7 ± 6.1
	VD	18.8 ± 5.9	18.3 ± 5.3	17.8 ± 4.4
Cortisol (nmol/L)	PLC	384.9 ± 111.1	320.5 ± 95.5	329.8 ± 92.7
	VD	389.9 ± 109.2	335.0 ± 106.1	320.6 ± 82.0
Growth hormone (mU/L)	PLC	0.777 ± 1.093	0.438 ± 0.341	0.744 ± 0.884
	VD	0.420 ± 0.690	0.339 ± 0.216	0.364 ± 0.264
IGF-1 (μg/L)	PLC	148.3 ± 32.9	149.0 ± 35.4	144.6 ± 27.6
	VD	131.5 ± 28.9	143.7 ± 34.7	132.8 ± 28.7
Insulin (mU/L)	PLC	8.75 ± 3.86	7.38 ± 2.99	8.61 ± 5.20
	VD	7.59 ± 2.94	7.74 ± 2.53	6.46 ± 2.99

Data are presented as means ± SD, *n* = 14 in PLC (placebo), and *n* = 14 in VD (vitamin D) group. IGF-1, insulin-like growth factor-1. * Statistically significant within-group difference compared to week 0 (*p* < 0.05).

**Table 8:** Serum cytokine levels during 12-week supplementation and resistance training period.

Variables	Placebo (*n* = 14)		Vitamin D (*n* = 10)	
	Week 0	Week 12	Week 0	Week 12
IL-1α (pg/mL)	0.09 ± 0.12	0.08 ± 0.13	0.05 ± 0.06	0.05 ± 0.08
IL-1β (pg/mL)	1.02 ± 0.71	1.06 ± 0.83	0.83 ± 0.24	0.75 ± 0.19
IL-4 (pg/mL)	1.08 ± 0.32	1.21 ± 0.48	1.07 ± 0.35	1.02 ± 0.32
IL-6 (pg/mL)	0.81 ± 0.58	0.80 ± 0.44	1.07 ± 0.67	1.11 ± 0.78
IL-8 (pg/mL)	10.20 ± 4.77	9.92 ± 4.92	11.24 ± 4.84	10.25 ± 3.90
IL-10 (pg/mL)	0.60 ± 0.43	0.55 ± 0.28	0.47 ± 0.18	0.42 ± 0.13
TNF-α (pg/mL)	2.68 ± 0.95	2.58 ± 1.11	2.44 ± 0.91	2.40 ± 0.65
IL-10/TNF-α	0.21 ± 0.09	0.21 ± 0.06	0.20 ± 0.08	0.18 ± 0.06
MCP-1 (pg/mL)	219.1 ± 60.7	225.3 ± 94.5	201.3 ± 93.2	196.9 ± 90.5

Data are presented as mean ± SD. IL, interleukin; TNF-α, tumor necrosis factor alpha; MCP-1, monocyte chemoattractant protein 1.

**Table 9:** Serum hemoglobin, ferritin, glucose concentrations, and HOMA-IR during 12-week supplementation and resistance training period.

Variable	Group	Week 0	Week 8	Week 12
Hemoglobin (g/L)	PLC	155.3 ± 6.7	154.2 ± 6.3	153.1 ± 5.4
	VD	150.2 ± 9.9	149.3 ± 8.1	148.1 ± 10.0
Ferritin (μg/L)	PLC	198.8 ± 106.0	166.8 ± 97.0	170.9 ± 99.2
	VD	190.0 ± 129.7	157.2 ± 109.3	164.3 ± 114.6
Glucose (mmol/L)	PLC	5.58 ± 0.60	5.44 ± 0.43	5.58 ± 0.44
	VD	5.55 ± 0.55	5.70 ± 0.29	5.54 ± 0.44
HOMA-IR	PLC	2.23 ± 1.22	1.79 ± 0.76	2.14 ± 1.30
	VD	1.88 ± 0.77	1.96 ± 0.66	1.60 ± 0.76

Data are presented as means ± SD, *n* = 14 in PLC (placebo), and *n* = 14 in VD (vitamin D) group.

**Table 10:** Serum metabolite concentrations and creatine kinase activity during 12-week supplementation and resistance training period.

Variable	Group	Week 0	Week 8	Week 12
Ionized calcium (mmol/L)	PLC	1.30 ± 0.06	1.28 ± 0.08	1.26 ± 0.05
	VD	1.27 ± 0.07	1.26 ± 0.08	1.27 ± 0.03
Calcium (mmol/L)	PLC	2.44 ± 0.08	2.43 ± 0.10	2.38 ± 0.09
	VD	2.38 ± 0.10	2.43 ± 0.04	2.41 ± 0.09
Urea (mmol/L)	PLC	6.09 ± 0.84	5.96 ± 0.88	6.18 ± 1.07
	VD	5.75 ± 1.21	6.68 ± 1.39 *	6.51 ± 1.47 *
Creatine kinase (U/L)	PLC	233.1 ± 174.1	170.6 ± 86.1	177.6 ± 72.2
	VD	152.4 ± 75.6	122.4 ± 44.0	169.4 ± 78.6

Data are presented as means ± SD, *n* = 14 in PLC (placebo), and *n* = 14 in VD (vitamin D) group. * Statistically significant within-group difference compared to week 0 (*p* < 0.05).

## Data Availability

The data presented in this study are available on request from the corresponding author (vahur.oopik@ut.ee).
